# Attenuation of Cigarette Smoke-Induced Airway Mucus Production by Hydrogen-Rich Saline in Rats

**DOI:** 10.1371/journal.pone.0083429

**Published:** 2013-12-20

**Authors:** Yunye Ning, Yan Shang, Haidong Huang, Jingxi Zhang, Yuchao Dong, Wujian Xu, Qiang Li

**Affiliations:** Department of Respiratory Medicine, Changhai Hospital, the Second Military Medical University, Shanghai, China; Institute of Lung Biology and Disease (iLBD), Helmholtz Zentrum München Germany

## Abstract

**Background:**

Over-production of mucus is an important pathophysiological feature in chronic airway disease such as chronic obstructive pulmonary disease (COPD) and asthma. Cigarette smoking (CS) is the leading cause of COPD. Oxidative stress plays a key role in CS-induced airway abnormal mucus production. Hydrogen protected cells and tissues against oxidative damage by scavenging hydroxyl radicals. In the present study we investigated the effect of hydrogen on CS-induced mucus production in rats.

**Methods:**

Male Sprague-Dawley rats were divided into four groups: sham control, CS group, hydrogen-rich saline pretreatment group and hydrogen-rich saline control group. Lung morphology and tissue biochemical changes were determined by immunohistochemistry, Alcian Blue/periodic acid-Schiff staining, TUNEL, western blot and realtime RT-PCR.

**Results:**

Hydrogen-rich saline pretreatment attenuated CS-induced mucus accumulation in the bronchiolar lumen, goblet cell hyperplasia, muc5ac over-expression and abnormal cell apoptosis in the airway epithelium as well as malondialdehyde increase in the BALF. The phosphorylation of EGFR at Tyr1068 and Nrf2 up-regulation expression in the rat lungs challenged by CS exposure were also abrogated by hydrogen-rich saline.

**Conclusion:**

Hydrogen-rich saline pretreatment ameliorated CS-induced airway mucus production and airway epithelium damage in rats. The protective role of hydrogen on CS-exposed rat lungs was achieved at least partly by its free radical scavenging ability. This is the first report to demonstrate that intraperitoneal administration of hydrogen-rich saline protected rat airways against CS damage and it could be promising in treating abnormal airway mucus production in COPD.

## Introduction

Chronic obstructive pulmonary disease (COPD) has become a major global epidemic that is increasing throughout the world, particularly in developing countries [1]. Goblet cell hyperplasia and excessive mucus production causes airway obstruction, which contributes to the morbidity and mortality of this disease [2]. Abnormal mucus production is now recognized as a key pathophysiological feature in COPD, including those without cough and sputum production and it should be a therapeutic target for all COPD subjects [3]. However, the therapies to target mucus effectively in asthma have either limits or no impact in COPD and they are not satisfactory to all COPD patients [4]. The development of safe and efficacious intervention for abnormal mucus production in COPD is still urgently needed.

Oxidant-antioxidant imbalance in lungs has been strongly implicated in COPD severity [5]. Oxidative stress increased in COPD patients [6] and chronic lung oxidative damage are key contributors to the pathogenesis of COPD, which includes mucus hypersecretion, heightened apoptosis and chronic inflammation [7]. Oxidative stress is considered to be an important therapeutic target in COPD [8]. Although some molecules such as N-acetylcysteine and its derivatives, which targeting mucin gel, can act as a precursor of reduced glutathione and as a direct reactive oxygen species (ROS) scavenger, and regulate the redox status in cells in COPD, unfortunately, sufficient blood concentrations of them are very difficult to achieve because of the fast turnover [9].

Hydrogen has been reported to selectively reduce hydroxyl radical and the most cytotoxicity of ROS. The reaction product is nothing else but water and might be safely applied in the clinic [10], [11]. In recent years, basic and clinical researches have shown that hydrogen-rich saline is efficacious in treating many disorders including oxygen toxicity, sepsis and hyperoxia- or ventilator-induced lung injury because of its antioxidant, anti-apoptotic, and anti-inflammatory properties [12]. Although Liu et al [13] hypothesized that hydrogen may be potentially effective for COPD by preventing its occurrence, exacerbation, and slowing its progress, it remains unknown if it has any effect on abnormal mucus production in COPD.

As cigarette smoking (CS) is the leading cause of COPD [14] and tracheal goblet cell hyperplasia as well as bronchoalveolar lavage fluid (BALF) mucin remained significantly elevated even when the rats were exposed to five cigarettes daily for 2 to 4 days [15], the current study was to investigate the effect of hydrogen-rich saline on CS-induced mucus production in rats.

## Materials and Methods

### Hydrogen-rich saline production and other reagents

Hydrogen was dissolved in physiological saline for 6 h under high pressure (0.4 MPa) to a supersaturated level. The saturated hydrogen-rich saline (400 ml) was freshly prepared in an aluminum bag, sterilized by gamma radiation and stored under atmospheric pressure at 4°C to maintain the concentration of hydrogen at higher than 0.6 mM. Gas chromatography was used to confirm the content of hydrogen in saline by the method described by Ohsawa et al [16].

Cigarettes were purchased from Guizhou Cigarette Factory (Brand Huangguoshu, Guizhou, China) (2.45 mg nicotine per cigarette, 40 mg/ml total particulate matter, nicotine content of 6%). Primary antibodies used were as follows: anti-muc5ac mouse monoclonal antibody (clone 45M1, Santa Cruz), anti-Nrf2 rabbit polyclonal antibody (Bioworld, USA), anti-total-EGFR rabbit polyclonal antibody (Proteintech, USA), anti-phospho-EGFR rabbit monoclonal antibody (Tyr1068) (Epitomic, USA) and HRP-anti-GAPDH (internal) antibody (Kangcheng, Shanghai, China). HRP-conjugated goat-anti-rabbit and rabbit-anti-mouse IgG were from Cell Signaling Technology (Beverly, USA).

### Ethics Statement

All animal experiments were performed in a humane manner, and also in accordance with the Institutional Animal Care Instructions. Animal handling and experimental procedures described herein were approved by the Ethical Committee on Animal Use of the Second Military Medical University. All animal manipulations were performed by trained personnel.

### Animals and treatment

Forty male Sprague-Dawley rats (180–200 g) were divided into four groups randomly with 10 rats each: sham control group (Control, Con), cigarette smoke group (CS), hydrogen-rich saline pretreatment group (CS+H), and hydrogen-rich saline control group (Hydrogen, H).

The rats were placed in 20 cm×40 cm×50 cm perspex chambers (5 rats/chamber) and exposed to cigarette smoke generated from 5 unfiltered cigarettes for 30 min, twice daily for 4 weeks according to a modified procedure based on the method as described [17]. Briefly, on each day of CS exposure, mainstream cigarette smoke from a burning cigarette was directly puffed into the exposure chamber by a 50 ml syringe and a special rubber catheter at a flow rate of 2 ml/s and a new cigarette was ignited after one burned up. The combustion time of a cigarette was 6 min. The CS total particulate matter (TPM) per cubic meter of air, which was used to monitor the smoke exposure (mean 450∼500 mg/m^3^ per second) in the chamber, was determined by a real-time aerosol dust monitor CEL-712 Microdust Pro (Casella, Bedford, UK). Rats in the hydrogen-rich saline pretreatment group received 10 ml/kg hydrogen-rich saline [18] intraperitoneally (i.p.) 30 min prior to CS exposure. Rats in the sham control or the hydrogen-rich saline control group were both exposed to air, but administrated i.p. with 10 ml/kg control saline or hydrogen-rich saline respectively each time.

All rats were housed in rooms maintained at constant temperature (21±2°C) and humidity (55±15%) with a 12-h light/dark cycle and allowed food and water ad libitum. The rats were anesthetized with an overdose of chloral hydrate i.p. followed by exsanguination 24 hours after the last treatment.

### Histopathology and immunohistochemistry

The right lung specimens of the rats were fixed in 10% formaldehyde for 24 hours, embedded in paraffin wax, and cut into 5-µm-thick sections which were stained with hematoxylin and eosin (H&E) to evaluate general morphology. The degrees of lung inflammation were evaluated by two analysts blinded to the groups using a subjective scale ranged from 0 to 4 (0, normal; 1, mild; 2, moderate; 3, severe; 4, very severe inflammation). For immunohistochemistry, the sections were immunostained with anti-muc5ac antibody. The sections were developed by diaminobenzidine (DAB) solution according to the manufacturer's instructions. Semi-quantitative analyses of the area of muc5ac-positive staining in the airway epithelium were defined by two independent investigators using the Image-Pro Plus program (Media Cybernetics) at a magnification of 200× by examining at least 50 consecutive fields for each group. Alcian Blue/periodic acid-Schiff (AB/PAS) staining was applied to detect acidic and neutral mucosubstances. Images of lung tissues with airways were captured by a Nikon microscope. AB/PAS-positive area and total area of corresponding bronchial epithelium were measured. Data were presented as the ratio of AB/PAS-positive area to the total area.

### Real-time RT-PCR

Total RNA was extracted from lung tissue homogenates using Trizol (Invitrogen), and real-time quantitative RT-PCR was performed by a Rotor-Gene 6000 real-time rotary analyzer (Corbett Research, Australia) using SYBR PCR Kit (Takara). PCR primers for *muc5ac* were, Forward: 5′-GCAATAACTCCACTTCCCTC-3′, Reverse: 5′-AGTCATAGCAGCATCCGTC-3′; Primers for *β-actin* were, Forward: 5′- TTACTGCCCTGGCTCCTAG-3′ Reverse: 5′-CGTACTCCTGCTTGCTGAT-3′. The transcription of *muc5ac* was normalized to that of *β-actin*.

### Western blot

Lung tissue homogenates were prepared with RIPA Buffer (Thermo Scientific) and 1 mM PMSF. Equal amount of denatured total protein (50 µg) was separated by 8% SDS-PAGE and transferred onto PVDF membranes. The membranes were incubated with appropriate dilution of primary antibodies against muc5ac, Nrf2, total-EGFR, phospho-EGFR (Tyr1068) and HRP-GAPDH respectively. After incubated with proper rabbit anti-mouse or goat anti-rabbit HRP-conjugated secondary antibodies, the intended proteins were detected using ECL kit (Invitrogen) and normalized to the corresponding GAPDH expression.

### In situ apoptosis assay

Terminal dUTP nick-end labeling (TUNEL) staining was performed on paraffin-embedded sections using the in situ cell death detection kit (Roche) according to the manufacturer's instructions to determine apoptosis in the airway epithelium. Apoptosis was manifested by brownish staining in the nuclei. Six microscopic fields (400 ×) of each section were randomly examined and the cells were counted by a single blinded observer in a coded randomized order. The apoptosis rate was presented as the percentage of TUNEL-positive cells to the total epithelial cells of corresponding airways from all rats in each group.

### Determination of malondialdehyde (MDA) content in the BALF

The left lungs of rats were repeatedly lavaged with 1 ml saline and the retrieval volume was maximized by compressing the thorax. BALF samples were stored at −80°C. MDA contents in the BALF were detected according to the protocol of a commercial kit purchased from Jiancheng Bioengineering Institute (Nanjing, China). Briefly, MDA in each sample reacted with Thiobarbituric Acid (TBA) to generate the MDA-TBA adduct. And then the MDA-TBA adduct was measured at 532 nm using a spectrophotometer (SmartSpec Plus, BIO-RAD, Hercules, CA). The levels of MDA was expressed as nmol/ml.

### Statistical analysis

Data from all the rats in each group are presented as mean±SD. One-way analysis of variance (ANOVA) was used to determine statistical significance between groups. Multiple comparisons were made by Student-Newman-Keuls post hoc test. p<0.05 were considered significant.

## Results

### Hydrogen-rich saline pretreatment protected CS-induced histopathological damages of rat lungs

**Figure 1:**
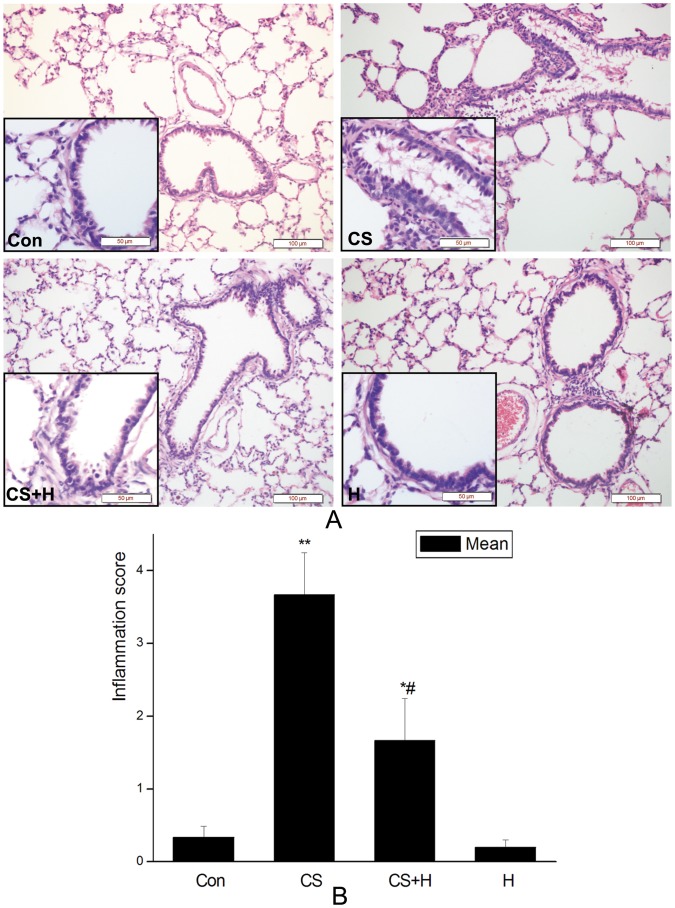
Effect of hydrogen on lung histopathology of rats exposure to CS. A. Representative H&E staining of lung sections. Con, sham control group; CS, cigarette smoke group; CS+H, hydrogen-rich saline pretreatment group; H, hydrogen-rich saline control group. (Scale bars  =  100 µm; lower-left insert: Scale bars  =  50 µm). B. Inflammation of rat lungs were scored. Hydrogen-rich saline significantly abrogated CS-induced lung inflammation. The results are presented as mean± SD (n = 10 rats per group) *p<0.05, **p<0.01 vs. the control group; #p<0.05 vs. the CS group.

Histopathological changes in rat airways were examined by H&E staining. After four consecutive weeks of repeated cigarette smoke exposure, bronchiolar lumen obstruction by mucus and cell debris, and inflammatory cell infiltration were observed in the lumen of lungs from rats in the CS exposure group. The inflammatory score of lungs from the CS exposure group was significantly higher than that from the control, but these changes induced by CS were effectively abrogated by hydrogen-rich saline pretreatment as shown in Figure 1.

### Hydrogen-rich saline inhibited CS-induced goblet cell hyperplasia in the rat airway

**Figure 2:**
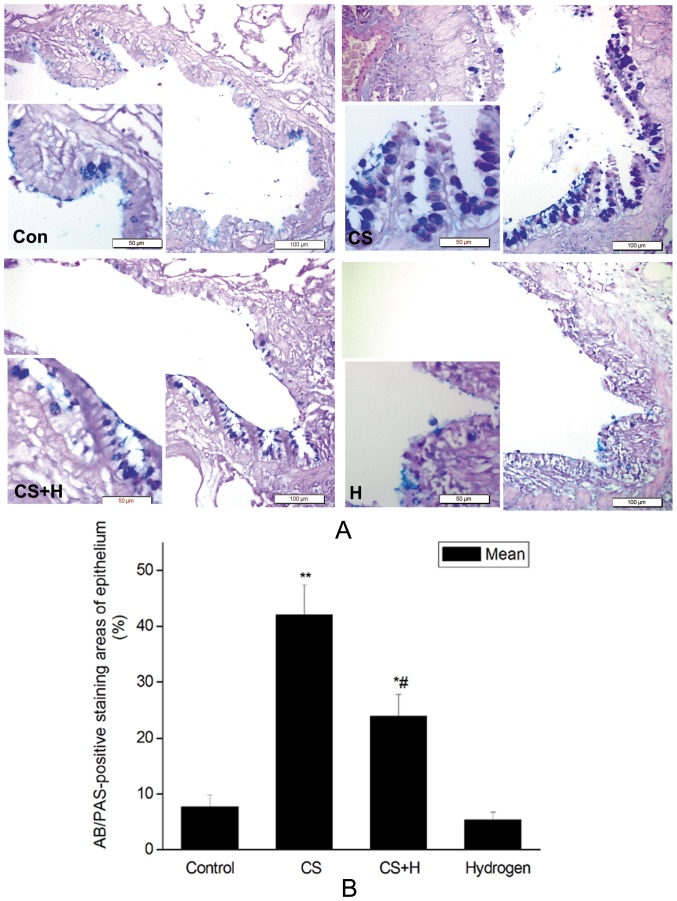
Effect of hydrogen on goblet cell hyperplasia of the bronchial epithelium detected by AB/PAS-staining. (A) Representative goblet cell staining determined by AB/PAS. Con, sham control group; CS, cigarette smoke group; CS+H, hydrogen-rich saline pretreatment group; H, hydrogen-rich saline control group. (Scale bars = 100 µm; lower-left insert: Scale bars = 50 µm); (B) Quantification of AB/PAS-positive area in the airway epithelium. AB/PAS-staining area and total area of corresponding bronchiolar epithelial were measured. AB/PAS-positive rates were presented as the ratio of AB/PAS-positive area to the total area. The values were expressed as mean ± SD from all the rats in each group.*p<0.05, **p<0.01 vs. Control, # p<0.05 vs. CS group.

Goblet cells, as determined by AB/PAS-staining and observed under the light microscopy, in the airway epithelium from CS-challenged rats contained large granular stores of AB-PAS-positive substances, while much lighter positive staining was observed in the hydrogen-rich saline pretreatment group (Figure 2A). Positive staining was sporadically seen in the epithelium of the sham control or the hydrogen-rich saline control rats. The positive rates of airway epithelium were 7.72±2.11%, 42.04±5.40%, 23.96±3.81% and 5.36±1.39% in the sham control, CS group, hydrogen pretreatment group and hydrogen control group respectively. CS exposure significantly increased AB/PAS-positive rate as compared with that in the control (p<0.01). As expected, hydrogen-rich saline significantly decreased CS-induced positive-staining rate as compared with that in the CS group (p<0.05) (Figure 2B).

### Hydrogen-rich saline inhibited CS-induced up-regulation of muc5ac expression

**Figure 3:**
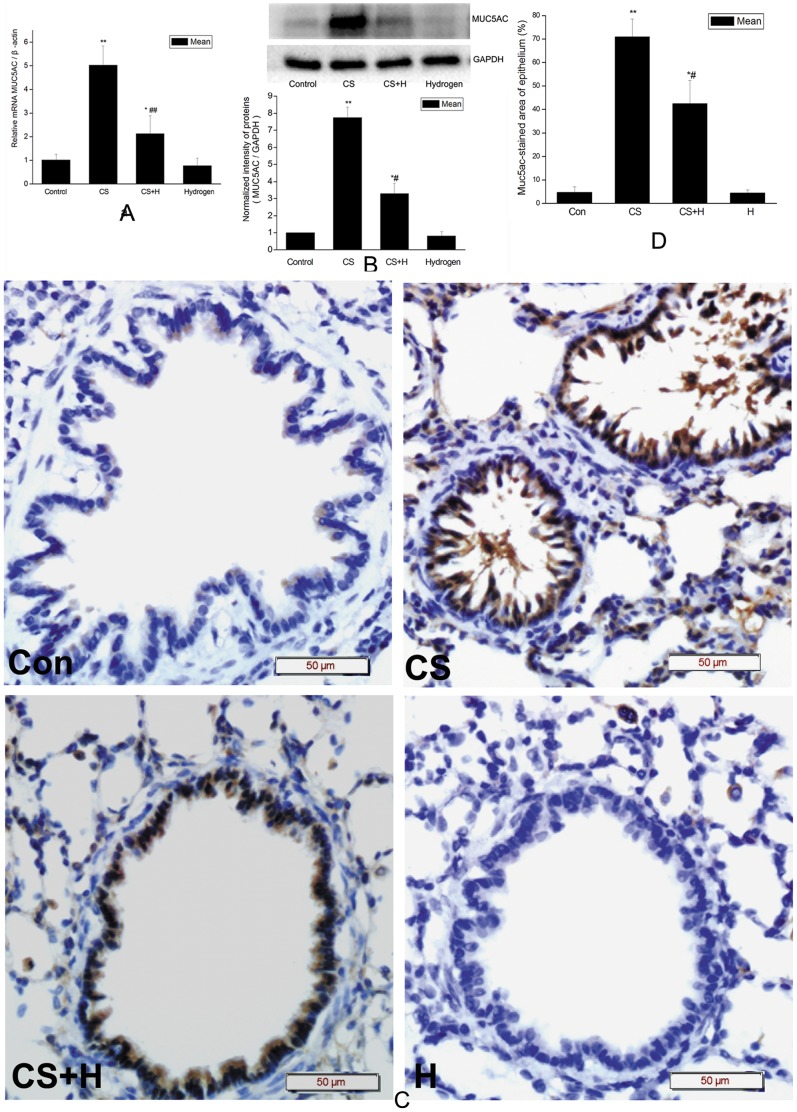
Effect of hydrogen on CS-induced *muc5ac* expression in rat lung tissues. (a) Effect of hydrogen on CS-induced *muc5ac* transcription analyzed by realtime RT-PCR. The levels of *muc5ac* mRNA were normalized to *β-actin*. Data are mean ±SD. * p<0.05, ** p<0.01 vs. the control; ## p<0.01 vs. CS group. (b) The upper panel was representative western blot analysis for muc5ac and GAPDH proteins in homogenized rat lung tissues. The bar graph (lower panel) showed muc5ac protein levels after normalized to the corresponding abundance of GAPDH. Data were presented as mean ± SD. *p<0.05, **p<0.01 vs. the control; # p<0.05 vs. CS group. (c) Representative immunohistochemistry for muc5ac in rat lung sections as indicated. Con, sham control group; CS, cigarette smoke group; CS+H, hydrogen-rich saline pretreatment group; H, hydrogen-rich saline control group. (Scale bars = 50 µm). Positive immunoreactivity for muc5ac was characterized by brown staining. (D) Percentage of Muc5ac-positive staining of the airway epithelium. Muc5ac-positive area and total area of corresponding bronchial epithelium were measured. Data were presented as the ratio of muc5ac-positive area to the total area. Hydrogen-rich saline pretreatment significantly decreased CS-induced muc5ac-positive area in the airway epithelium. The values were expressed as mean± SD (n = 10 per group). *p<0.05, **p<0.01 vs. the control, # p<0.05 vs. the CS group.

To determine the effect of hydrogen-rich saline on CS-induced expression of *muc5ac* gene, the main gel-forming mucin, realtime RT-PCR, western blot, and IHC were applied to detect the mRNA and protein levels in the rat lungs respectively. As shown in Figure 3A, the level of *muc5ac* mRNA increased by about 5 fold in the rat lungs of CS exposure group as compared with that in the control group (p<0.01). Hydrogen-rich saline significantly decreased CS-induced *muc5ac* mRNA level (p<0.01 vs. CS group).

Meanwhile, western blot results demonstrated muc5ac protein in the lung tissue homogenates from the CS exposure group was nearly 8 fold higher than that from the control (p<0.01). Hydrogen-rich saline significantly inhibited CS-challenged up-regulation of muc5ac expression (p<0.05 vs. CS group) (Figure 3B).

Immunohistochemical analysis demonstrated that positive immunoreactivity for muc5ac antibody in the lung tissue especially in the airway epithelium from CS exposure rats was characterized by brown staining, while hydrogen alleviated the positive staining of muc5ac both in the alveolar wall and airway epithelium (Figure 3C). Semi-quantitative analyses of IHC demonstrated that the percentage of bronchial epithelium muc5ac-positive area significantly increased in the CS exposure group as compared with that in the control (p<0.01), and this increase was significantly abrogated by hydrogen-rich saline (p<0.05 vs. CS group) (Figure 3D).

Together, these results suggested CS up-regulated *muc5ac* gene expression at both mRNA and protein levels while hydrogen-rich saline attenuated this up-regulation in rat lungs.

### Hydrogen-rich saline attenuated CS-induced airway epithelial cell apoptosis in rats

**Figure 4:**
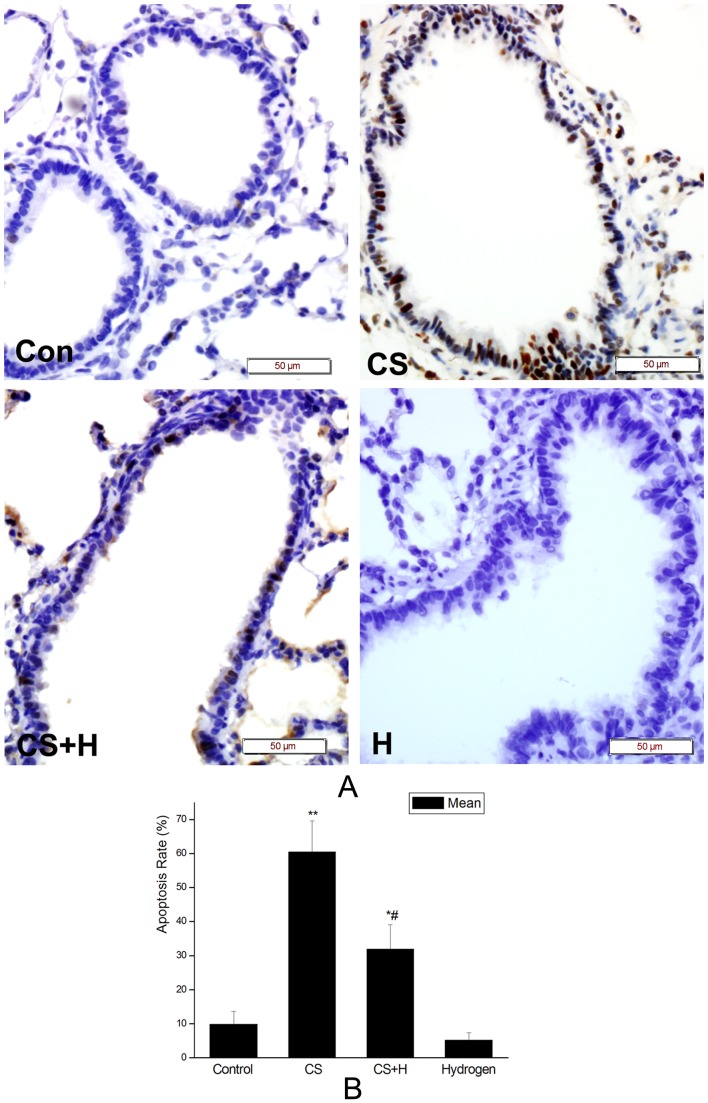
Effect of hydrogen on CS-induced airway epithelial cell apoptosis in rats. Representative TUNEL staining in small airways. Con, sham control group; CS, cigarette smoke group; CS+H, hydrogen-rich saline pretreatment group; H, hydrogen-rich saline control group. (Scale bars = 50 µm). (B) The bar graph showed the apoptosis rate of airway epithelial cells in each group as indicated. Data were presented as mean ± SD of the apoptosis rate from all the rats in each group as indicated. *p<0.05, **p<0.01 vs. the control; #p<0.05 vs. CS group.

As shown in Figure 4A, CS stimulated airway epithelial cell apoptosis while hydrogen-rich saline protected the rat lungs against CS-induced abnormal cell apoptosis.

The apoptosis rate of airway epithelial cells in CS exposure rats were 60.50±9.12%, which was significantly higher than that in the control (9.83±3.87%) (p<0.01). After intervened by hydrogen-rich saline, the rate of TUNEL-positive cells in the airway epithelium reduced to 32.0±7.07% (p<0.05 vs. the CS group). While hydrogen-rich saline alone had little effect on the apoptosis rate of airway epithelial cells (5.17±2.23%) (Figure 4B).

### Alleviation of oxidative damage may be a critical factor in the event that hydrogen inhibited CS-induced airway mucus production

**Figure 5:**
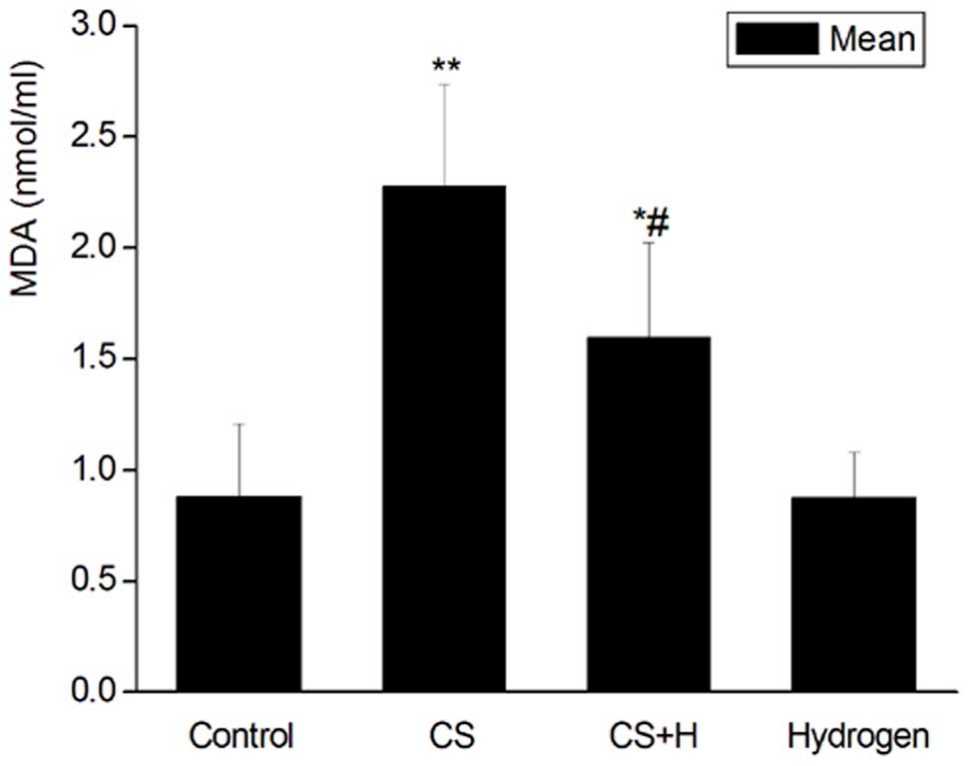
Effect of hydrogen on CS-induced MDA production in the BALF of rats. MDA contents in the BALF were determined using a chemical reaction kit. Data were expressed as mean ± SD, n = 10 for each group.*p<0.05, **p<0.01 vs. Control. #p<0.05 vs. the CS group.

To explore if hydrogen-rich saline inhibited CS-induced mucus production through its ability to scavenge free radicals, we detected the level of MDA in the BALF to evaluate the change of CS-induced oxidative damage. As shown in Figure 5, the content of MDA in the BALF from the CS group (2.28±0.46 nmol/ml) was significantly higher than that from the sham control (0.88±0.33 nmol/ml) (p<0.01). The MDA level in the BALF from the hydrogen pretreatment group (1.59±0.43 nmol/ml) decreased significantly as compared with that from the CS group (p<0.05). Hydrogen alone had little effect on the production of MDA in the BALF (0.87±0.21 nmol/ml).

### EGFR signaling cascade and oxidative stress signaling might be involved in the effect of hydrogen on CS-induced airway mucus production

**Figure 6:**
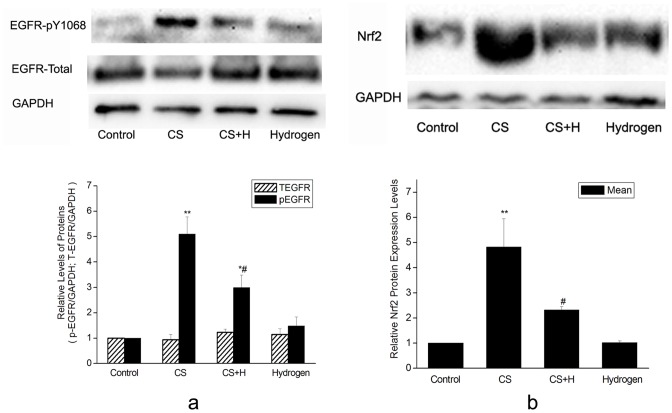
Effect of hydrogen on CS-induced phosphorylation of EGFR and expression of Nrf2. (a) Representative photographs of western blot for EGFR, p-EGFR (Tyr1068) proteins from homogenized rat lungs were shown in the upper panel. EGFR and p-EGFR(Tyr1068) protein levels were normalized to the corresponding GAPDH as shown in the lower bar graph. (b) The upper panel was representative western blot analysis for Nrf2 protein from homogenized rat lung tissues as indicated. The level of Nrf2 protein was normalized to the corresponding GAPDH as shown in the lower panel. Data were presented as mean ± SD from all rats in each group as indicated. *p<0.05, **p<0.01 vs. Control; #p<0.05 vs. CS group.

To further explore the mechanism how hydrogen-rich saline intervened CS-induced mucus production, we detected the phosphorylation of EGFR at Tyr1068, an important active site of EGFR, and the expression of Nrf2 protein, an important downstream element of oxidative stress signaling. The results showed that CS up-regulated phospho-EGFR at Tyr1068 by 5.1 fold. Hydrogen-rich saline attenuated CS-induced phosphorylation of EGFR by 51.33 percent. While total EGFR protein remained unchanged in each group ( Figure 6A).

As shown in Figure 6B, the Nrf2 protein level increased significantly in the CS-challenged rat lungs as compared with that in the control (p<0.01), while hydrogen-rich saline pretreatment resulted in a significant reduction of Nrf2 protein as compared with that in the CS group (p<0.05). This revealed that hydrogen-rich saline protected rat lungs against CS damage at least in part by its potent free radical scavenging ability.

## Discussion

Effective blockade of abnormal mucus production in COPD will reduce hospitalizations, morbidity and mortality, and long-term control of mucus may lessen the burden of this disease [19]. In the present study, we demonstrated that hydrogen ameliorated *muc5ac* gene expression and goblet cell hyperplasia induced by cigarette smoke, the principal aetiology of COPD. To the best of our knowledge, this is the first report to imply intraperitioneal administration of hydrogen-rich saline protected lungs from CS exposure damage in a rat model.

Mucus is composed of water, ions, lipids, proteins, and complex macromolecular glycoproteins called mucins, which render viscoelastic and gel-forming properties to mucus [20]. Airway mucus plays an important role in host defense mechanisms as a physicochemical barrier to inhaled particles and gases, bacteria and viruses, but over-production of mucus is harmful, which is a distinguishing feature of chronic inflammatory airway diseases [21]. Mucus hyperproduction is commonly associated with goblet cell hyperplasia in the airway epithelium. In the present study, the results that goblet cell hyperplasia and mucus plugs in the bronchiolar lumen in chronic CS exposure rats were alleviated by hydrogen-rich saline demonstrated that hydrogen was efficacious against CS-induced abnormal airway mucus. Muc5ac and muc5b are major gel-forming mucins that are responsible for the biophysical properties of the mucus. Muc5b is constitutively expressed, while muc5ac is inducible from the goblet cells of airway epithelium [22]. Hydrogen-rich saline could down-regulate CS-induced muc5ac expression at both transcriptional and translational levels, as is consistent with the goblet cell hyperplasia. These results revealed hydrogen has the potential to treat mucus hypersecretion in COPD, however, much remains to be done to clarify the effect of hydrogen-rich saline on this disease.

It was reported that increased cell apoptosis occurred in the bronchial/bronchiolar epithelium of rats exposed to mainstream cigarette smoke [23]. In the present study abnormal apoptosis of airway epithelial cells was also observed in the lungs from chronic CS exposure rats, but CS-induced cell apoptosis was inhibited by hydrogen-rich saline. This demonstrated the protective role of hydrogen-rich saline on CS-challenged rat lungs.

CS delivers and generates free radicals, which orchestrate the inflammation, mucous gland hyperplasia, and apoptosis of the airway epithelium within the lungs [24]. Given that hydrogen could serve as a potent free radical scavenger by exclusively quenching ROS, particularly hydroxyl radical, the most devastating reactive oxygen species [16], we have explored whether hydrogen intervened the effect of CS on rat lungs due to its antioxidant ability. The results showed the level of MDA, a common indicator of oxidative damage to membrane lipid, in the BALF from the CS-challenged rats increased and hydrogen-rich saline significantly inhibited its propagation. This revealed that hydrogen inhibited CS-induced goblet cell proliferation and muc5ac expression at least partly due to its ROS scavenging activity to reduce CS-induced oxidative damage. It was reported that hydrogen has the potential to easily diffuse into organelles of cultured cells and no known toxic effects on human body [25]. Patients benefited from the antioxidant intervention of hydrogen by drinking H_2_-loaded water in clinical trials [26], [27]. These revealed the possible clinical importance of hydrogen in treating CS-induced abnormal mucus production.

Oxidative stress occurs when ROS are produced in excess of the antioxidant defense mechanisms and cannot be scavenged in time. Mammalian cells produce several antioxidant enzymes to defend them against oxidative damage. The transactivation of the majority antioxidant and defense genes is regulated by Nrf2. Cigarette smoke, which contains a variety of oxidants, can activate Nrf2 [28]. In this study, hydrogen down-regulating the expression of CS-induced Nrf2 further implied that hydrogen protected rat lungs against damage from CS exposure by its antioxidant ability.

EGFR signaling activation played a critical role in oxidative stress-induced goblet cell hyperplasia and muc5ac up-regulation expression [29]. Rats exposed to cigarette smoke up to 4 weeks showed increased p-EGFR-Tyr1068 protein levels in the lungs, while this phosphorylation activation was abrogated by hydrogen intervention. These results revealed that the inhibition of EGFR cascade was implicated in the effect of hydrogen-rich saline on CS-stimulated mucus production.

In conclusion, hydrogen protected airway epithelium from CS damage and abrogated CS-induced airway mucus production in rats. This protective role of hydrogen-rich saline on CS-exposed rat lungs was achieved at least partly by its antioxidant ability and the inhibition of Nrf2 and EGFR signaling pathway might be implicated in this process. Since inhaled hydrogen gas at therapeutic dose has no adverse effects on the saturation level of arterial oxygen and hemodynamic parameters [30], hydrogen-rich saline, safer and more convenient than hydrogen gas, could be promising in treating mucus hypersecretion in COPD.

## References

[1] Barnes PJ (2007) Chronic obstructive pulmonary disease: a growing but neglected global epidemic. PLoS Med 4: e112. 17503959 10.1371/journal.pmed.0040112 PMC1865560

[2] Fahy JV, Dickey BF (2010) Airway mucus function and dysfunction. N Engl J Med 363: 2233–2247. 21121836 10.1056/NEJMra0910061 PMC4048736

[3] Burgel PR, Martin C (2010) Mucus hypersecretion in COPD: should we only rely on symptoms? European Respiratory Review 19: 94–96. 20956176 10.1183/09059180.00004410 PMC9682577

[4] Sivaprasad U, Askew D, Ericksen M, Gibson A, Stier M, et al.. (2010) A non-redundant role for Serpinb3a in the induction of mucus production in asthma. J Immunol 184: 141.117–. 10.1016/j.jaci.2010.10.009 PMC3058372 21126757

[5] MacNee W (2005) Pulmonary and Systemic Oxidant/Antioxidant Imbalance in Chronic Obstructive Pulmonary Disease. Proceedings of the ATS 2: 50–60. 10.1513/pats.200411-056SF 16113469

[6] Repine J, Bast A, Lankhorst IDA (1997) The Oxidative Stress Study Group (1997) Oxidative Stress in Chronic Obstructive Pulmonary Disease. Am J Respir Crit Care Med 156: 341–357. 9279209 10.1164/ajrccm.156.2.9611013

[7] Malhotra D, Thimmulappa R, Navas-Acien A, Sandford A, Elliott M, et al (2008) Decline in NRF2-regulated Antioxidants in Chronic Obstructive Pulmonary Disease Lungs Due to Loss of Its Positive Regulator, DJ-1. Am J Respir Crit Care Med 178: 592–604. 18556627 10.1164/rccm.200803-380OC PMC2542433

[8] MacNee W, Rahman I (1999) Oxidants and Antioxidants as Therapeutic Targets in Chronic Obstructive Pulmonary Disease. Am J Respir Crit Care Med 160: S58–65. 10556172 10.1164/ajrccm.160.supplement_1.15

[9] Sadowska AM (2012) N-Acetylcysteine mucolysis in the management of chronic obstructive pulmonary disease. Therapeutic Advances in Respiratory Disease 6: 127–135. 22361928 10.1177/1753465812437563

[10] Nakao A, Sugimoto R, Billiar TR, McCurry KR (2009) Therapeutic antioxidant medical gas. J Clin Biochem Nutr 44: 1–13. 19177183 10.3164/jcbn.08-193R PMC2613492

[11] Hardeland R (2012) Hydrogen therapy: a future option in critical care? Crit Care Med 40: 1382–1383. 22425856 10.1097/CCM.0b013e318241112a

[12] Huang CS, Kawamura T, Toyoda Y, Nakao A (2010) Recent advances in hydrogen research as a therapeutic medical gas. Free Radic Res 44: 971–982. 20815764 10.3109/10715762.2010.500328

[13] Liu SL, Liu K, Sun Q, Liu WW, Tao HY, et al (2011) Hydrogen Therapy may be a Novel and Effective Treatment for COPD. Front Pharmacol 2: 19. 21687512 10.3389/fphar.2011.00019 PMC3108576

[14] Mannino DM, Buist AS (2007) Global burden of COPD: risk factors, prevalence, and future trends. Lancet 370: 765–773. 17765526 10.1016/S0140-6736(07)61380-4

[15] Stevenson CS, Coote K, Webster R, Johnston H, Atherton HC, et al (2005) Characterization of cigarette smoke-induced inflammatory and mucus hypersecretory changes in rat lung and the role of CXCR2 ligands in mediating this effect. Am J Physiol Lung Cell Mol Physiol 288(3): L514–522. 15516486 10.1152/ajplung.00317.2004

[16] Ohsawa I, Ishikawa M, Takahashi K, Watanabe M, Nishimaki K, et al (2007) Hydrogen acts as a therapeutic antioxidant by selectively reducing cytotoxic oxygen radicals. Nat Med 13: 688–694. 17486089 10.1038/nm1577

[17] Chen L, Sun BB, Wang T, Wang X, Li JQ, et al (2010) Cigarette smoke enhances β-defensin 2 expression in rat airways via nuclear factor-κ B activation. European Respiratory Journal 36: 638–645. 20150208 10.1183/09031936.00029409

[18] Sun Q, Cai J, Liu S, Liu Y, Xu W, et al (2011) Hydrogen-rich saline provides protection against hyperoxic lung injury. J Surg Res 165: e43–49. 21067781 10.1016/j.jss.2010.09.024

[19] Curran DR, Cohn L (2010) Advances in Mucous Cell Metaplasia: A Plug for Mucus as a Therapeutic Focus in Chronic Airway Disease. Am J Respir Cell Mol Biol 42: 268–275. 19520914 10.1165/rcmb.2009-0151TR PMC2830403

[20] Voynow JA, Gendler SJ, Rose MC (2006) Regulation of Mucin Genes in Chronic Inflammatory Airway Diseases. Am J Respir Cell Mol Biol 34: 661–665. 16456183 10.1165/rcmb.2006-0035SF

[21] Shimizu T, Hirano H, Shimizu S, Kishioka C, Sakakura Y, et al (2001) Differential Properties of Mucous Glycoproteins in Rat Nasal Epithelium. A Comparison between Allergic Inflammation and Lipopolysaccharide Stimulation. Am J Respir Crit Care Med 164: 1077–1082. 11588000 10.1164/ajrccm.164.6.2012058

[22] Perez-Vilar J, Mabolo R, McVaugh CT, Bertozzi CR, Boucher RC (2006) Mucin Granule Intraluminal Organization in Living Mucous/Goblet Cells: ROLES OF PROTEIN POST-TRANSLATIONAL MODIFICATIONS AND SECRETION. J Biol Chem 281: 4844–4855. 16377632 10.1074/jbc.M510520200

[23] D'Agostini F, Balansky RM, Izzotti A, Lubet RA, Kelloff GJ, et al (2001) Modulation of apoptosis by cigarette smoke and cancer chemopreventive agents in the respiratory tract of rats. Carcinogenesis 22(3): 375–380. 11238175 10.1093/carcin/22.3.375

[24] Lin JL, Thomas PS (2010) Current perspectives of oxidative stress and its measurement in chronic obstructive pulmonary disease. COPD 7(4): 291–306. 20673039 10.3109/15412555.2010.496818

[25] Rahman I, van Schadewijk AAM, Crowther AJL, Hiemstra PS, Stolk J, et al (2002) 4-Hydroxy-2-Nonenal, a Specific Lipid Peroxidation Product, Is Elevated in Lungs of Patients with Chronic Obstructive Pulmonary Disease. Am J Respir Crit Care Med 166: 490–495. 12186826 10.1164/rccm.2110101

[26] Kajiyama S, Hasegawa G, Asano M, Hosoda H, Fukui M, et al (2008) Supplementation of hydrogen-rich water improves lipid and glucose metabolism in patients with type 2 diabetes or impaired glucose tolerance. Nutr Res 28: 137–143. 19083400 10.1016/j.nutres.2008.01.008

[27] Nakao A, Toyoda Y, Sharma P, Evans M, Guthrie N (2010) Effectiveness of hydrogen rich water on antioxidant status of subjects with potential metabolic syndrome-an open label pilot study. J Clin Biochem Nutr 46: 140–149. 20216947 10.3164/jcbn.09-100 PMC2831093

[28] Yageta Y, Ishii Y, Morishima Y, Masuko H, Ano S, et al (2011) Role of Nrf2 in Host Defense against Influenza Virus in Cigarette Smoke-Exposed Mice. J Virol 85: 4679–4690. 21367886 10.1128/JVI.02456-10 PMC3126158

[29] Casalino-Matsuda SM, Monzon ME, Forteza RM (2006) Epidermal Growth Factor Receptor Activation by Epidermal Growth Factor Mediates Oxidant-Induced Goblet Cell Metaplasia in Human Airway Epithelium. Am J Respir Cell Mol Biol 34: 581–591. 16424381 10.1165/rcmb.2005-0386OC PMC2644222

[30] Hayashida K, Sano M, Ohsawa I, Shinmura K, Tamaki K, et al (2008) Inhalation of hydrogen gas reduces infarct size in the rat model of myocardial ischemia-reperfusion injury. Biochem Biophys Res Commun 373: 30–35. 18541148 10.1016/j.bbrc.2008.05.165

